# Postoperative awake prone position in geriatric patients with hip fractures: a protocol for a randomized controlled trial on the efficacy of postoperative prone position in reducing pulmonary complications and improving oxygenation

**DOI:** 10.1186/s13063-023-07308-x

**Published:** 2023-04-18

**Authors:** Yu-cheng Gao, Liu Shi, Yuan-wei Zhang, Wang Gao, Xie Tian, Mu-min Cao, Ying-juan Li, Hui Chen, Yun-feng Rui

**Affiliations:** 1 Department of Orthopaedics, Zhongda Hospital, School of Medicine, Southeast University, No. 87 Ding Jia Qiao, Nanjing, Jiangsu 210009 People’s Republic of China; 2 Orthopaedic Trauma Institute (OTI), Southeast University, No. 87 Ding Jia Qiao, Nanjing, Jiangsu 210009 People’s Republic of China; 3 Trauma Center, Zhongda Hospital, School of Medicine, Southeast University, No. 87 Ding Jia Qiao, Nanjing, Jiangsu 210009 People’s Republic of China; 4 Multidisciplinary Team (MDT) for Geriatric Hip Fracture Comprehensive Management, Zhongda Hospital, School of Medicine, Southeast University, No. 87 Ding Jia Qiao, Nanjing, Jiangsu 210009 People’s Republic of China; 5 Department of Geriatrics, Zhongda Hospital, School of Medicine, Southeast University, No. 87 Ding Jia Qiao, Nanjing, Jiangsu 210009 People’s Republic of China

**Keywords:** Hip fracture, Geriatric, Awake prone position, Postoperative pulmonary complications, Hypoxemia

## Abstract

**Introduction:**

Postoperative pulmonary complications (PPCs) are prevalent in geriatric patients with hip fractures. Low oxygen level is one of the most important risk factors for PPCs. Prone position has been proven efficacy in improving oxygenation and delaying the progress of pulmonary diseases, especially in patients with acute respiratory distress syndrome induced by multiple etiologies. The application of awake prone position (APP) has also attracted widespread attention in recent years. A randomized controlled trial (RCT) will be carried out to measure the effect of postoperative APP in a population of geriatric patients undergoing hip fracture surgery.

**Methods:**

This is an RCT. Patients older than 65 years old admitted through the emergency department and diagnosed with an intertrochanteric or femoral neck fracture will be eligible for enrollment and assigned randomly to the control group with routine postoperative management of orthopedics or APP group with an additional prone position for the first three consecutive postoperative days (PODs). Patients receiving conservative treatment will not be eligible for enrollment. We will record the difference in the patient’s room-air-breathing arterial partial pressure of oxygen (PaO_2_) values between the 4^th^ POD (POD 4) and emergency visits, the morbidity of PPCs and other postoperative complications, and length of stay. The incidence of PPCs, readmission rates, and mortality rates will be followed up for 90 PODs.

**Discussion:**

We describe the protocol for a single-center RCT that will evaluate the efficacy of postoperative APP treatment in reducing pulmonary complications and improving oxygenation in geriatric patients with hip fractures.

**Ethics and dissemination:**

This protocol was approved by the independent ethics committee (IEC) for Clinical Research of Zhongda Hospital, Affiliated to Southeast University, and is registered on the Chinese Clinical Trial Registry. The findings of the trial will be disseminated through peer-reviewed journals.

**Ethics approval number:**

2021ZDSYLL203-P01

**Trial registration:**

ChiCTR ChiCTR2100049311. Registered on 29 July 2021.

**Trial status:**

Recruiting. Recruitment is expected to be completed in December 2024.

**Supplementary Information:**

The online version contains supplementary material available at 10.1186/s13063-023-07308-x.

## Introduction

Given the aging population, the increasing number of geriatric hip fractures has become a tremendous health burden for society globally [1, 2]. For such patients, active surgical treatment has been proven optimal compared with conservative treatment [3]. Geriatric patients are characterized by fragility and multi-comorbidities, which brings a challenge for perioperative management and preoperative rehabilitation [4]. Postoperative complications are always inevitable and impact considerably on the prognosis of geriatric hip fractures [5, 6], among which, postoperative pulmonary complications (PPCs) were the commonest one and the incidence rate was up to 12.6% [7–11]. The PPCs are considered a highly heterogeneous disease group [12]. It usually brought extended length of stays (LOS), increased health care costs, readmission, and even mortality [12–15]. Low oxygen level was one of the independent risk factors of PPCs [16]. Postoperative hypoxemia was persistent and more common than in the preoperative period [17]. Canet et al. pointed out that the morbidity of PPCs would increase significantly if preoperative arterial oxygen saturation (SaO_2_) was less than 90% [13]. Russotto et al. also discovered that the morbidity of postoperative pneumonia (POP) was in liner negative correlation with preoperative SaO_2_ [14]. Moreover, arterial partial pressure of oxygen (PaO_2_) level was a simple predictor of POP with high acceptance in geriatric hip fracture patients and the cut-off value was 72.5 mmHg, which met the standard of hypoxemia (PaO_2_<80mmHg) [18–20].

Nowadays, prone position ventilation was widely applicated in adjuvant therapy for acute respiratory distress syndrome (ARDS), especially secondary to coronavirus disease 2019 (COVID-19), and recommended in the international guideline for the management of critical COVID-19 patients [21]. Recently, prone positioning in non-intubated or awake prone position (APP) characterized by a simple mechanism of action and perceived low risk has also received lots of interest and brought positive effects [22].

For geriatric patients with hip fractures, no widely accepted, long-term, feasible intervention with provable evidence to treat PPCs has been proposed [23, 24]. Lying in the lateral position intra-operatively could improve postoperative PaO_2_/ fraction of the inspired oxygen (FiO2) (P/F) and reduce the need for invasive or non-invasive ventilation in patients with femoral neck fracture (FNF), which remained us change of body position may help to better respiratory function via gas exchange for such patients [25]. We hypothesize that the application of the APP will reduce the risk of PPCs and improve postoperative oxygenation (expressed as P/F) in geriatric patients with hip fractures, an ideal population for randomized controlled trials (RCT).

### Objective

To investigate the hypothesis that the APP would reduce the incidence of PPCs and elevate the room-air-breathing P/F in geriatric patients with hip fractures.

## Method and analysis

### Overview of trial design

**Fig. 1:**
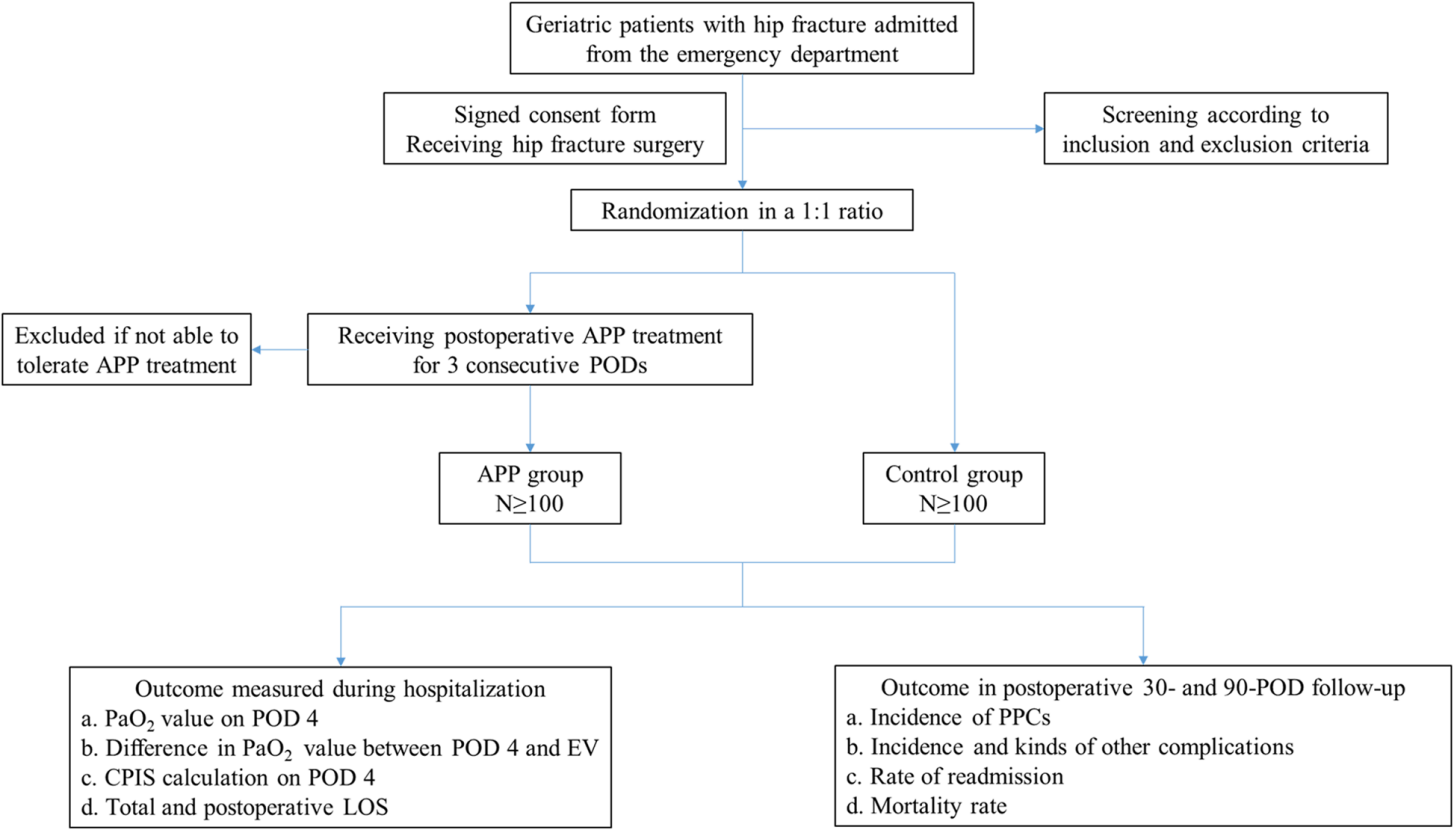
Flow chart of study design. *APP* awake prone position, *POD* postoperative day, *PaO*_*2*_ arterial partial pressure of oxygen, *EV* emergency visits, *LOS* length of stay, *PPCs* postoperative pulmonary complications, *CPIS* clinical pulmonary infection score

**Fig. 2:**
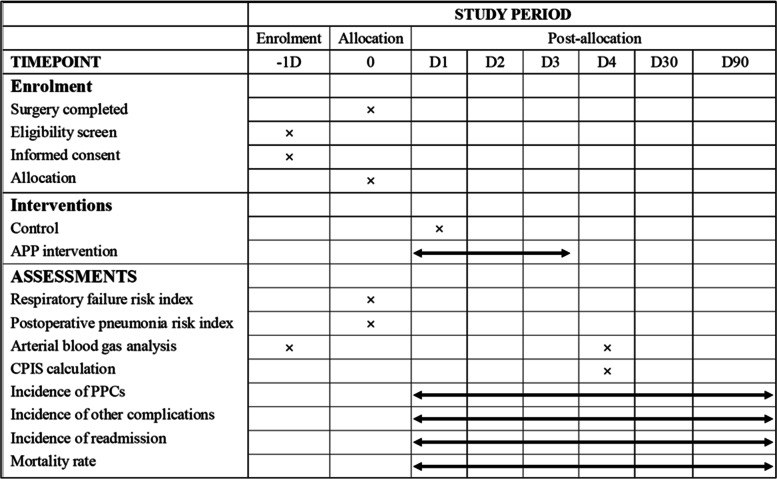
Schedule of enrolment, interventions, and assessments

We will conduct a single-center RCT using a completely randomized two-arm design to investigate whether APP can decrease the morbidity of PPCs and improve postoperative oxygenation for geriatric patients with hip fractures (Figs. 1 and 2). The Standard Protocol: Items: Recommendations for Interventional Trials (SPIRIT) checklist [26] is provided as Additional file 1. All patients considered for inclusion are admitted from the emergency department to the Trauma and Orthopedic Unit of the Zhongda Hospital affiliated to Southeast University and will be operated by the same team in a fixed operating room. A designated investigator will employ a 1:1 randomization system to assign patients who meet the criteria to the APP group and the control group. Patients enrolled in the study will be followed for at least 90 PODs.

Patients will undergo a review of hematological parameters including routine blood, biochemistry, electrolyte, and blood gas analysis on POD 4. Clinical pulmonary infection score (CPIS) [27] (Additional file 2) will be calculated on POD 4. The incidence of various postoperative complications, mortality rate, and LOS during hospitalization will be also recorded. At the same time, the incidence of postoperative complications especially PPCs, readmission, and mortality rates will be calculated within 30 and 90 PODs, respectively. This protocol has been approved by the Independent Ethics Committee (IEC) for Clinical Research of Zhongda Hospital, Affiliated to Southeast University (2021ZDSYLL203-P01), and is registered on the Chinese Clinical Trial Registry (ChiCTR2100049311). Only the head of this series of research (YR) has the right to make the final decision to terminate the trial if any serious adverse event judged to be related to the study treatment occurs.

### Primary research question

In geriatric patients with hip fractures, will the addition of APP to routine postoperative treatment reduce the risk of developing PPCs?

### Secondary research question

In geriatric patients with hip fractures, will the addition of APP to routine postoperative treatment result in:

1. Improved postoperative oxygenation?
2. Shorter LOS?
3. Decreased incidence of other kinds of postoperative complications, readmission, and mortality rates?

### Hypothesis

Compared with the control group, geriatric patients with hip fractures who receive postoperative APP treatment will have a lower incidence of PPCs and an improved postoperative oxygenation.

### Setting and participants

Geriatric patients (over 65 years old) presenting to the emergency department with an acute hip fracture from September 1, 2021, to December 31, 202,4 will be admitted to the Trauma and Orthopedic Unit of the Zhongda Hospital affiliated to Southeast University. Before admission, trauma center physicians will take the patient's medical history, risk assessment, physical examination, and necessary laboratory and imaging tests. According to inclusion and exclusion criteria, patients will be screened for eligibility and enrolled as potential participants by one investigator (YG).

Once confirmed to receive surgical treatment, one research implementer (YG) will obtain informed consent from potential participants to take part in the study by means of conversation and sign the approved IEC informed consent forms. All patients will understand that depending on the randomization results, they may or may not receive a postoperative APP. The total number of hospitalized patients who meet the inclusion and exclusion criteria exceeds the minimum sample size required for the study. However, some patients may hesitate to participate in the study due to concerns about the safety of APP and follow-up support of the related unexpected events. To attract more potential participants for the study, we will inform patients of the following during the conversation: firstly, the application of APP after hip fracture surgery in elderly patients has been shown to be safe in the previous attempt; secondly, APP may have a positive effect on preventing postoperative complications and accelerating postoperative recovery; finally, in the event of any unexpected adverse events, the hospital will assume full responsibility for resolution. Participants in the study are voluntary, and if they refuse, their decision will not affect any other aspect of their care. Patients will have the right to learn any details of the intervention and to withdraw from the study at any point.

### Inclusion criteria

Patients meeting the following criteria will be considered for eligibility:

1. Aged over 65
2. Informed consent and voluntary participation in a clinical trial
3. Diagnosed with acute hip fracture induced by low-energy injury including intertrochanteric fracture and FNF within 14 days before admission
4. In at least one of the following conditions:
  a Type 2 diabetes mellitus (T2DM)
  b Chronic lung disease such as chronic obstructive pulmonary disease
  c Pre-injury activities of daily living (ADL) score < 60
  d Modified British Medical Research Council (mMRC) score ≥ 2
  e First result of leukocyte count > 10 × 10^9^/L or C-reaction protein (CRP) > 15 mg/L at emergency visits (EV)
  f First result of PaO_2_ < 80 mmHg or SaO_2_ < 96% at EV
  g First result of hemoglobin < 100 g/L at EV
  h First result of serum albumin < 35 g/L at EV
  i Previous history of smoking and sleep apnea syndrome

5. Able to tolerant the prone position for at least 30 min one time
6. Intertrochanteric fracture patients receiving closed reduction and internal fixation (proximal femoral locking gamma nail); FNF patients receiving posterior approach uncemented total hip arthroplasty (THA) or hemiarthroplasty (HHA) as procedure methods

### Exclusion criteria

Patients meeting the following criteria will be considered for exclusion.

1. With life-threatening organ dysfunction, or unable to disengage from guardianship or non-invasive/invasive oxygen supplement
2. With pulmonary comorbidities which have the same name as sub-diseases of PPCs before surgery
3. With osteosarcoma, multiple myeloma, and various other pathological fractures
4. With multiple fractures or severe vascular, nerve, or soft tissue injury injuries
5. With a bilateral hip fracture
6. Receiving hip revision surgery
7. With a history of major cardiac or pulmonary surgery
8. In an immune-disorder condition caused by various causes (malignant tumors, severe autoimmune diseases, long-term hormone use, etc.)

### Baseline data

Baseline data will include gender, age, body mass index, type of fracture, time from injury to surgery, pre-injury ADL score, mMRC score, American Society of Anesthesiologists classification, comorbidities including pulmonary disease according to the first result of thoracic computerized tomography (CT) at admission (atelectasis, pleural effusion, chronic obstructive pulmonary disease), hypertension, T2DM, coronary atherosclerotic heart disease, cognitive dysfunction, prior stroke, cerebral infarction, history of smoking and drinking, first laboratory results of arterial blood gas analysis [PaO_2_, PaCO_2_, SaO_2_, pH, lactic acid], routine blood test (erythrocyte count, leukocyte count, CRP, hemoglobin), biochemistry test (serum albumin, blood glucose, blood creatinine, blood urea nitrogen), electrolyte test (serum potassium, serum sodium), D-dimer, brain natriuretic peptide (BNP) at EV, time from injury to surgery, proportion of receiving surgery within 48 h after admission, surgical procedures (closed reduction and internal fixation, THA or HHA), methods of anesthesia (general or epidural anesthesia), postoperative respiratory failure risk index [28] (Additional file 3), POP risk index [29] (Additional file 4), duration of surgery, perioperative blood transfusion volume.

### Randomization and allocation

Randomization will take place once participants have signed informed consent for the surgical procedure. SAS software 9.4 will be used to generate the random table as an allocation sequence by an investigator (LS) and random numbers will be assigned by the envelope method. Based on random numbers, another investigator (TX) will assign patients to the APP group or control group in a 1:1 ratio. Details of any planned restriction will be provided in a separate document that is unavailable to those who enroll participants or assign interventions.

### Blinding

Keeping trial participants and care providers blinded is unrealistic. But neither the outcome assessors nor the data analysts are aware of the grouping. Grouping information will be represented to data analysts in the form of group A/B rather than the APP group and control group. During the data analysis phase, all personal information that can be used to identify the participants will be hidden.

### Intervention

After signing informed consent, participants will be enrolled and randomly assigned to either the APP group or the control group. Surgical treatment will be performed on an emergency basis upon completion of the necessary pre-operative preparations. The surgical procedures, including closed reduction and internal fixation, THA, and HHA, will be decided by the chief physician (HC and YR) in trauma orthopedics. All operations will be scheduled in a fixed operating theatre and completed by the same team. Patients in the APP group will receive the prone position for at least half an hour at a time, and at least once per day for the first three consecutive PODs. The first APP treatment each day will be guided and supervised. The frequency and duration of other spontaneous prone positions of patients in the APP group will not be constrained. Except for this, during this period, all patients will receive the same postoperative management including postoperative oxygen inhalation (2L/min) for 6 h, standardized nutritional support, anti-inflammatory, detumescent, analgesic, hypodermic anti-coagulant prophylaxis, and regular atomization therapy. Both FNF and intertrochanteric fracture patients will roll onto their broken side into a prone position. Prone position pads will be used to suspend the upper abdomen and chest to reduce intra-abdominal and intra-thoracic pressure, allowing the alveoli to expand sufficiently. If severe adverse events such as unbearable pain, suspected prosthesis dislocation or internal fixation failure, chest tightness, and difficulty breathing occur, or the patients report that he/she cannot tolerate the prone position, the intervention should be stopped immediately. The investigators’ department will treat and compensate the patient for any additional injuries resulting from APP treatment. Intervention time as an adherence quantitative indicator and the reasons for intervention termination will be recorded in the database. Hematological parameters and chest radiography will be re-examined on the morning of POD 4. Patients will be followed up for at least 90 PODs through outpatient visits or telephone calls. In addition to orthopedic-related tests, they will undergo chest CT in an outpatient setting to determine if PPCs are present. The conduct of the trial will be audited weekly, independently of the investigators.

*Standard of discharge*: (1) The x-ray shows that the position of the internal fixation/prosthesis is in place. (2) The incision is essentially healed, and no signs of infection are found. (3) No serious complications have occurred requiring continued hospitalization. (4) The serological test results and the general condition of the patient are acceptable.

### Primary outcome

The primary outcome of the study will be the incidence of PPCs during the first 30 PODs. PPCs include all related diseases (atelectasis, POP, ARDS, pulmonary embolism, pleural effusion, pneumothorax, cardiogenic pulmonary edema, and bronchospasm) diagnosed anywhere.

### Secondary outcome

The secondary outcomes will be PaO_2_ on POD 4, the difference in PaO_2_ between POD 4 and EV, CPIS on POD 4, LOS, other types of postoperative complications, readmission, and mortality in 90-POD follow-up.

Given that FiO_2_ is equal to 20.9% of the breathing room air, we will use PaO_2_ instead of P/F to reflect the patient’s oxygenation capacity. On the morning of POD 4, the patient will undergo arterial blood gas analysis while inhaling room air. We anticipate that postoperative oxygenation (expressed as PaO_2_), will be higher in the APP group than in the control group. To minimize the impact of the baseline oxygenation capacity of different participants on the results, we will use the difference in PaO_2_ between POD 4 and EV to balance out this individual difference. Additional tests required for the diagnosis of complications, such as chest CT, pulmonary and coronary CT angiography, brain CT, arterial blood gas analysis, myocardial X-ray, troponin, and BNP, will be performed as soon as suspicious symptoms such as dyspnea, chest distress, fever, cough, sputum, and motor, sensory or cognitive dysfunction appear. All post-operative complications will be diagnosed following consultation with the appropriate specialist. Readmission and mortality rates within 30 or 90 PODs will be calculated separately. All serious adverse events, as well as all non-serious adverse events that are unexpected and determined to be related to the APP treatment, will be recorded in the study database and reported on request to the IEC for Clinical Research of Zhongda Hospital, Affiliated to Southeast University.

### Sample size consideration

According to the result of the pilot study, the sample size will be 200 (100 in the APP group and 100 in the control group respectively) if taking the incidence of PPCs within the first 30 PODs as the primary outcome and 20%the as loss rate [30].

### Data collection and analysis plan

All data will be collected and stored in case report form by two well-trained assessors (WG, MC) independently. Any discrepancies will be checked by the same two assessors to ensure accuracy and authenticity. All information will be aggregated in the final trial dataset, which will be accessible only to LS and will only be shared with research team members upon request for the completion of the designated learning task. All information related to patient identification will be hidden before being uploaded to a public database. Descriptive analyzes of the patient population will include reporting means (with SDs) for normally distributed variables, medians (with Q1, Q3) for skew-distributed variables, and frequencies (with percentages) for categorical variables. Student t-tests will be conducted to analyze normally distributed variables. The Mann-Whitney *U* test will be used for skew-distributed data and ordered categorical variables. Chi-squared tests will be used to analyze binary or unordered categorical variables. The balance of baseline data and differences in postoperative outcomes will be assessed in terms of the above variables. To control other covariates, including stratification factors (gender and fracture type) and baseline variables that have potential relation with the outcome, as well as any such variable that appears imbalanced between the APP group and control group, multiple logistic regression model will be conducted to analyze the related factors of PPCs at each point of time (POD 30 and 90). Similarly, we will perform a multivariate linear regression analysis to determine which variables are independently related to the difference in PaO_2_ between POD 4 and EV. All analyses will be performed using the SPSS 23.0 software. Any outcome data collected for participants who discontinued or deviated from the intervention protocol will be listed. Primary and secondary outcome analysis will not allow missing data. Other missing values will be assumed to show no significant difference from other data we have collected; If missing values suggest that the missingness is not random, we will assess the potential impact of missing data on our results via a developmental model.

### Patient and public involvement

Patients and the public will not be involved in the design, conduct, reporting, or dissemination plans of the research.

## Discussion

### PPCs in geriatric patients with hip fracture

Prolonged bed rest, acute lung injury (ALI) induced by trauma, airway abrasion and oedema caused by endotracheal intubation, shallow respiration, and insufficient ventilation resulted from anesthetic and muscle relaxant residue all contributed to the occurrence of PPCs [31]. The prevalence of PPCs in geriatric patients with hip fractures was the highest among postoperative complications, reaching up to 12.6% [7–11]. However, the existing clinical treatment measures are not satisfactory. So, we are urgently looking for more suitable and effective adjuvant therapies, and if a simple, feasible, invasive, and inexpensive measure can benefit such patients, it would be worth considering.

PPCs can be divided into two categories, one group members with the same pathophysiological mechanisms of airway collapse and contamination, including atelectasis, POP, respiratory failure, and ARDS, and the other group members without a common physiological mechanism, which should be evaluated separately, such as pulmonary embolism, pleural effusion, pneumothorax, cardiogenic pulmonary edema, and bronchospasm [32]. Among the subtypes of PPCs following hip fracture surgery in the elderly, POP and respiratory failure are the major components and are always considered severe [33]. The incidence of POP is up to 3.5~15.2% [34] and could bring a 30-day mortality rate up to 27–43% in geriatric patients with hip fractures [35, 36]. Postoperative respiratory failure is significantly associated with preoperative hypoxemia, which is highly prevalent in geriatric patients with hip fractures [37]. Given this, we will specifically evaluate the risk of POP and respiratory failure in participants to further determine the comparability of the two groups before intervention.

### The physiological viewpoint of the prone position

To avoid fracture displacement and the extreme pain caused by stimulation of pulled periosteum after injury, a supine position was used for injured limb braking. In the supine position, due to gravity, the compression of the heart and mediastinum on the lower lobe of the lung and the mismatch between the spherical thoracic cavity and the triangular lung led to compressive atelectasis. The alveoli, especially the dorsal alveoli, are limited in expansion, resulting in a mismatch of ventilation-perfusion ratio and relative shunt fraction, and ultimately the decline of oxygenation index [38, 39]. Prone position can reduce the pressure gradient of the vertical pleura and the compression of the lung lobe to preferentially distribute lung perfusion in the dorsal region, reduce local alveolar abnormalities resulting from increased tension and stress changes, and limit the overexpansion of healthy areas, and eventually improve lung hemodynamics, optimize the pulmonary ventilation blood flow ratio, reduce airway resistance, improve oxygenation index, promote alveolar expansion, reduce the risk of infection [39–43].

### Clinical application of prone position treatment

Previous researches have shown that prone position ventilation may reduce the incidence of ventilator-associated pneumonia, but does not reduce mortality in patients with acute respiratory failure and may increase the risk of other complications such as pressure ulcers [44, 45].

However, there still existed a growing body of clinical evidence supporting that the prone position is of positive significance in delaying the progress of pulmonary diseases and improving prognosis. Compared with the left lateral position, patients who underwent thoracoscopic esophagectomy in the prone position had a markedly higher oxygenation index after the surgery, though no significance was found in the PPCs between the two surgical positions [46]. Prone position has been proven to effectively reduce the risk of mortality and length of intensive care unit stay in ventilator-associated pneumonia patients [47]. Moreover, as one of the protective lung ventilation strategies, the prone position could reduce mortality in severe ARDS and acute respiratory failure patients significantly [48–51]. When applicated to conscious patients, the prone position manifested a similar outcome. Early APP combined with noninvasive ventilation or high-flow nasal canula could reduce or delay the need for intubation and the possibility to develop critical patients in moderate to severe ARDS patients with COVID-19 [52, 53]. For acute hypoxia patients, APP was recommended to be carried out early to prevent the worsening of hypoxemia and respiratory failure [41].

### Prone position in geriatric patients with hip fracture

If there exists evidence from both anatomic physiology theory and clinical practice that the prone position can improve pulmonary function, we believe that its application should not be limited to patients with severe ARDS. Whether pulmonary complications, which are common following fracture, can be prevented by prone position treatment arouses our interest. Unfortunately, so far, to the best of our knowledge, there have been no reports of the prone position treatment being used in the orthopedic field. This trial is an attempt to extend the application of APP to the postoperative management of geriatric patients with hip fractures in the general ward and provide high-quality evidence.

We believe that postoperative APP treatment in elderly patients with hip fractures is feasible according to the following facts: (1) The only absolute contraindications for prone ventilation were unstable rib or spinal fractures [42]. (2) The development and maturity of anti-rotary intramedullary nail and hip joint arthroplasty technology realize the possibility of early pain relief and rehabilitation activities after hip fracture surgery. (3) No possible complications such as prosthesis dislocation and internal fixation failure occurred in 42 patients arranged in the APP group in our preliminary trials. (4) Improving pulmonary function and physiological status via postural change is better than relying on exogenous oxygen supplements. Moreover, inappropriate exogenous oxygen supplementation could further damage alveolar type II epithelial cells and might cause refractory hypoxemia and respiratory failure [54].

We choose to carry out APP treatment for the first three consecutive PODs to prevent PPCs for the following reasons: 1. The peak time for diagnosis of PPCs and hypoxemia occurred on POD 4 after hip fracture surgery in the elderly [10, 55]. 2. The early postoperative hyperinflammatory state would lead to ALI and increase the risk of pulmonary infection. Animal experiments have shown that elderly rats exhibited the highest pulmonary inflammation level histologically in the first three days following hip fracture fixation. 3. In the first three PODs, the pulmonary infection risk and mortality rate of elderly rats receiving hip fracture fixation reached a peak if inoculating in the airway with Pseudomonas aeruginosa before surgery [56–59].

As reported, the duration of the therapeutic prone position was at least up to 2 h per day was considered to be effective [60]. However, as a preventive measure for PPCs, the suitable duration of APP is still uncertain. According to the previous attempt, the tolerance limitation for one time to keep a prone position in the first 3 consecutive PODs for most patients receiving hip fracture surgery was not longer than 30 min. Furthermore, we have no indication to use sedative drugs for conscious patients in the general ward to prolong the time in a prone position as if in the intensive care unit, which is unethical. According to the preliminary trial results, there were significant differences in some outcomes between the APP group and control group, such as the difference in PaO_2_ between POD 4 and EV, and the incidence of PPCs within the first 30 PODs. If the results of the RCT prove the efficacy of postoperative APP in geriatric patients with hip fractures, this research could share our experience and help develop subsequent implementation procedures.

## Trial status

The trial opened to recruitment in September 2021 and will close to recruitment in December 2024 (protocol version 20230220).

## Supplementary Information

**Additional file 1.** SPIRIT Checklist for Trials.

**Additional file 2.** Clinical pulmonary infection score (CPIS).

**Additional file 3.** Postoperative respiratory failure risk index.

**Additional file 4.** Postoperative pneumonia risk index.

## Data Availability

Not applicable - no identifying images or other personal or clinical details of participants are presented here or will be presented in reports of the trial results. The participant information materials and informed consent form are available from the corresponding author on request. Protocol amendments Any important protocol modifications will be communicated with investigators, REC/IRBs, trial participants, trial registries, journals, and regulators.

## Abbreviations

ADL: Activities of daily living
ALI: Acute lung injury
APP: Awake prone position
ARDS: Acute respiratory distress syndrome
BNP: Brain natriuretic peptide
COVID-19: Coronavirus disease 2019
CPIS: Clinical pulmonary infection score
CRP: C-reaction protein
CT: Computerized tomography
EV: Emergency visits
FNF: Femoral neck fracture
FiO2: Fraction of the inspired oxygen
HHA: Hemiarthroplasty
IEC: Independent ethics committee
LOS: Length of stay
mMRC: Modified British Medical Research Council
PaCO2: Arterial partial pressure of carbon dioxide
PaO2: Arterial partial pressure of oxygen
P/F: PaO2/FiO2
PODs: Postoperative days
POP: Postoperative pneumonia
PPCs: Postoperative pulmonary complications
RCT: Randomized controlled trial
SaO2: Arterial oxygen saturation
SPIRIT: Standard Protocol: Items: Recommendations for Interventional Trials
THA: Total hip arthroplasty
T2DM: Type 2 diabetes mellitus
