# Supplementary material for: Postoperative awake prone position in geriatric patients with hip fractures: a protocol for a randomized controlled trial on the efficacy of postoperative prone position in reducing pulmonary complications and improving oxygenation
Source: Trials. 2023 Apr 18;24:280. doi: 10.1186/s13063-023-07308-x (PMC10110345; doi:10.1186/s13063-023-07308-x)
Supplement: Supplementary file 2 — Additional file 2. Clinical pulmonary infection score (CPIS). [file 13063_2023_7308_MOESM2_ESM.docx]

| Clinical pulmonary infection score (CPIS) calculation | |
| --- | --- |
| Parameter | Value |
| Temperature (℃) |  |
| ≥36.5 and ≤38.4 | 0 |
| ≥38.5 and ≤38.9 | 1 |
| ≥39 or ≤36 | 2 |
| Blood leukocytes (mm3) |  |
| ≥4000 and ≤11000 | 0 |
| ＜4000 or ＞11000 | 1 |
| + band forms ≥50% | Add 1 |
| Tracheal secretion |  |
| Absence of tracheal secretions | 0 |
| Presence of non-purulent tracheal secretions | 1 |
| Presence of purulent tracheal secretions | 2 |
| Oxygenation: PaO2/FIO2(mmHg) |  |
| >240 or ARDS | 0 |
| ≤240 and no ARDS | 1 |
| Pulmonary radiography |  |
| No infiltrate | 0 |
| Diffuse (or patchy) infiltrate | 1 |
| Localized infiltrate | 2 |
| Progression of pulmonary infiltrate |  |
| No radiographic progression | 0 |
| Radiographic progression (after CHF and ARDS excluded) | 1 |
| Culture of tracheal aspirate |  |
| Pathogenic bacteria cultured in rare or light quantity or no growth | 0 |
| Pathogenic bacteria cultured in moderate or heavy quantity | 1 |
| Same pathogenic bacteria seen on Gram stain | Add 1 |
