# Supplementary material for: Postoperative awake prone position in geriatric patients with hip fractures: a protocol for a randomized controlled trial on the efficacy of postoperative prone position in reducing pulmonary complications and improving oxygenation
Source: Trials. 2023 Apr 18;24:280. doi: 10.1186/s13063-023-07308-x (PMC10110345; doi:10.1186/s13063-023-07308-x)
Supplement: Supplementary file 3 — Additional file 3. Postoperative respiratory failure risk index. [file 13063_2023_7308_MOESM3_ESM.docx]

| RESPIRATORY FAILURE RISK INDEX | |
| --- | --- |
| Preoperative Predictor | Point Value |
| Type of surgery |  |
| Abdominal aortic aneurysm | 27 |
| Thoracic | 21 |
| Neurosurgery, upper abdominal, or peripheral vascular | 14 |
| Neck | 11 |
| Emergency surgery | 11 |
| Albumin (<30 g/L) | 9 |
| Blood urea nitrogen (>30 mg/dL) | 8 |
| Partially or fully dependent functional status | 7 |
| History of chronic obstructive pulmonary disease | 6 |
| Age |  |
| >=70 | 6 |
| 60-69 | 4 |
