# Supplementary material for: Postoperative awake prone position in geriatric patients with hip fractures: a protocol for a randomized controlled trial on the efficacy of postoperative prone position in reducing pulmonary complications and improving oxygenation
Source: Trials. 2023 Apr 18;24:280. doi: 10.1186/s13063-023-07308-x (PMC10110345; doi:10.1186/s13063-023-07308-x)
Supplement: Supplementary file 4 — Additional file 4. Postoperative pneumonia risk index. [file 13063_2023_7308_MOESM4_ESM.docx]

| Postoperative Pneumonia Risk Index | |
| --- | --- |
| Preoperative Risk Factor | Point Value |
| Type of surgery |  |
| Abdominal aortic aneurysm repair | 15 |
| Thoracic | 14 |
| Upper abdominal | 10 |
| Neck | 8 |
| Neurosurgery | 8 |
| Vascular | 3 |
| Age |  |
| >80 | 17 |
| 70–79 | 13 |
| 60-69 | 9 |
| 50-59 | 4 |
| Functional status |  |
| Totally dependent | 10 |
| Partially dependent | 6 |
| Weight loss >10% in past 6 months | 7 |
| History of chronic obstructive pulmonary disease | 5 |
| General anesthesia | 4 |
| Impaired sensorium | 4 |
| History of cerebrovascular accident | 4 |
| Blood urea nitrogen level |  |
| <2.86 mmol/L (<8 mg/dL) | 4 |
| 7.85–10.7 mmol/L (22–30 mg/dL) | 2 |
| >=10.7 mmol/L (>=30 mg/dL) | 3 |
| Transfusion >4 units | 3 |
| Emergency surgery | 3 |
| Steroid use for chronic condition | 3 |
| Current smoker within 1 year | 3 |
| Alcohol intake >2 drinks/d in past 2 weeks | 2 |
